# Heterogeneity of porcine bone marrow-derived dendritic cells induced by GM-CSF

**DOI:** 10.1371/journal.pone.0223590

**Published:** 2019-11-05

**Authors:** Sang Eun Kim, Jeong Ho Hwang, Young Kyu Kim, Hoon Taek Lee

**Affiliations:** 1 Department of Animal Biotechnology, Konkuk University, Gwangjin-gu, Seoul, Republic of Korea; 2 Animal Model Research Group, Jeonbuk Department of Inhalation Research, Korea Institute of Toxicology, Jeongeup, Jeollabuk-do, Republic of Korea; 3 Department of Stem Cell and Regenerative Biotechnology, Konkuk University, Gwangjin-gu, Seoul, Republic of Korea; Temple University, Lewis Katz School of Medicine, UNITED STATES

## Abstract

*In vitro* generation of dendritic cells (DCs) is advantageous for overcoming the low frequency of primary DCs and the difficulty of applying isolation techniques for studying DC immunobiology. The culture of bone marrow cells with granulocyte-macrophage colony-stimulating factor (GM-CSF) has been used extensively to generate bone marrow-derived dendritic cells (BMDCs). Studies have reported the heterogeneity of cells grown in murine GM-CSF culture based on the levels of MHCII expression. Although porcine DCs are generated by this classical method, the exact characteristics of the BMDC population have not yet been defined. In this study, we discriminated GM-CSF-grown BMDCs from gnotobiotic miniature pigs according to several criteria including morphology, phenotype, gene expression pattern and function. We showed that porcine BMDCs were heterogeneous cells that differentially expressed MHCII. MHCII^high^ cells displayed more representative of DC-like morphology and phenotype, including costimulatory molecules, as well as they showed a superior T cell priming capacity as compared to MHCII^low^ cell. Our data showed that the difference in MHCII^high^ and MHCII^low^ cell populations involved distinct maturation states rather than the presence of different cell types. Overall, characterization of porcine BMDC cultures provides important information about this widely used cellular model.

## Introduction

Dendritic cells (DCs) are components of the immune system that can present antigens to T cells [1]. Conventional DCs (cDCs) provide signals for T cell activation and differentiation, and are therefore regarded as professional antigen-presenting cells (APCs) of the immune system [2]. However, study of these essential cells has been complicated by the low frequency of DC populations in blood and tissue. For this reason, the biology of DCs has been studied in cells grown *in vitro* from hematopoietic precursors, in the presence of growth factors [3]. Besides, *in vitro* generated DCs have been designated as cell-based vaccines for immunotherapy [4]. Bone marrow cells (BMCs) have been cultured with granulocyte-macrophage colony-stimulating factor (GM-CSF), a cytokine involved in the development and homeostasis of mononuclear phagocytes, to generate bone marrow-derived dendritic cells (BMDCs) that resemble tissue DC [5, 6].

In bone marrow cultures induced by GM-CSF, CD11c^+^ MHCII^+^ cells have been assumed to be the source of pure BMDCs, whereas macrophages are thought to be adherent cells [3, 7]. However, the studies reported that this classical method produces heterogeneous populations of murine myeloid cells in non-adherent populations and loosely adherent populations [8–10]. The study suggested that MHCII^high^ cells, which were previously shown to be DCs and MHCII^low^ cells, closely resemble macrophages in the murine GM-CSF-derived heterogeneous population. Other studies suggested that MHCII^low^ cells contain immature DCs, which further upregulate MHCII on their surface, indicating maturation in mice [11, 12].

The porcine immune system is similar to the human immune system with respect to DC biology [13, 14]. The gnotobiotic miniature pig is the best model to study immunology, including immune cell ontogeny, microbial infection, and xenotransplantation [15–18]. To study porcine DC biology, *in vitro* differentiated DCs have been widely used [19], especially BMCs are cultured with GM-CSF for generation of BMDCs likewise other species [20]. The non-adherent cells have been considered as pure BMDCs and are characterized by expression of the surface molecules, CD1, CD16, CD80/86, CD172a, and MHC class II [21]. However, it is unclear whether porcine BMDCs are heterogeneous like murine BMDCs.

In this study, BMCs were isolated from gnotobiotic miniature pigs and cultured with GM-CSF to generate DCs. We classified GM-CSF-grown porcine BMDCs into MHCII^high^ cells and MHCII^low^ cells, in a similar manner as murine BMDCs. These two populations from non-adherent cells were characterized according to their morphology, phenotype, gene expression profile, and function. On the basis of these characteristics, we showed that non-adherent cells isolated from GM-CSF-grown BMC cultures were heterogeneous in terms of their levels of MHCII expression. Therefore, these findings of GM-CSF-derived porcine BMDCs could lead to improvements in our understanding of the porcine immune system.

## Materials and methods

### Animals

Gnotobiotic miniature pigs were kept under absolute barrier contained facility at the Bio-organ Research Center of Konkuk University, Seoul, Republic of Korea [22]. Animal experiments were carried out based on the National Institutes of Health guidelines for the care and use of laboratory animals. The study was conducted after obtaining approval from the Institutional Animal Care and Use Committee (IACUC) of Konkuk University (KU16168). In this study, three, 3-week-old piglets were used: K8082-1, K8082-2, and K8083-4. The animals were sacrificed using CO_2_ according to IACUC guidelines, and then the humerus, tibia, and femur were collected to isolate BMCs.

### Cell preparation

The BMDCs were generated using a previously described method with some modifications [20]. The BMCs were cultured for 10 days at a density of 5 × 10^5^ cells/mL in RPMI-1640 medium (Gibco, Gaithersburg, MD, USA) supplemented with 10% heat-inactivated fetal bovine serum (FBS; Gibco), 100 U/mL penicillin, 100 μg/mL streptomycin, 1 mM minimal essential medium, non-essential amino acids (Gibco), and 100 ng/mL of porcine GM-CSF (R&D Systems, Madrid, Spain). GM-CSF was additionally supplemented on days 2, 4, and 6. Differentiated cells were obtained from the non-adherent cell population after day 10. The cells were incubated at 37°C in a humidified atmosphere of 5% CO_2_ in air.

### Flow cytometry analysis

The cells were incubated with monoclonal antibody from hybridoma culture supernatants for 30 min, and then washed twice with Dulbecco’s phosphate-buffered saline (DPBS; Welgene, Seoul, Republic of Korea). The cells were incubated with secondary antibody and washed twice with DPBS. Fluorescein isothiocyanate (FITC)-conjugated goat anti-mouse IgG (Biolegend, San Diego, CA, USA) and allophycocyanin (APC)-conjugated goat anti-mouse IgG (Biolegend) were used as secondary antibodies. The cells were resuspended in 500 μL fluorescence-activated cell sorter buffer (5% FBS in DPBS) and flow cytometry analysis was performed on a BD Accuri^™^ C6 flow cytometer (Becton Dickinson, Franklin Lakes, NJ, USA). The following antibodies were used: anti-porcine CD1 (HB140), anti-porcine CD172a (HB142), anti-porcine CD16 (G7), anti-porcine CD11b/CD18 (PM3-15), and anti-porcine MHCII (MSA3) from hybridoma culture supernatants. Anti-porcine CD14 ascites, APC-conjugated anti-human CD86 (IT2.2; Biolegend), APC-conjugated anti-human CD163 (GHI/61; Biolegend), and anti-porcine CD117 (2B8/BM; Bio-Rad, Hercules, CA, USA) were also used.

To isolate MHCII^high^ and MHCII^low^ populations from BMDCs and c-kit^+^ hematopoietic stem cells (HSCs) from BMCs, the cells were sorted by a FACSAria^™^ instrument (Becton Dickinson). Flow cytometry analysis was conducted using FlowJo software (https://www.flowjo.com/).

### RNA sequencing

Total RNA was extracted from sorted cell subsets including c-kit^+^ HSC, MHCII^high^, and MHCII^low^ cells using TRIzol reagent (Invitrogen, Carlsbad, CA, USA). In order to construct cDNA libraries with the TruSeq RNA library kit (illumine, San Diego, CA, USA), 1ug of total RNA was used. The protocol consisted of polyA-selected RNA extraction, RNA fragmentation, random hexamer primed reverse transcription and 100nt paired-end sequencing by Illumina HiSeq2500 (illumine, San Diego, CA, USA). The libraries were quantified using qPCR according to the qPCR Quantification Protocol Guide and qualified using an Agilent Technologies 2100 Bioanalyzer (Agilent, Santa Clara, CA, USA). We processed reads from the sequencer and aligned them to the *Sus scrofa* using Tophat v2.0.13 [23]. Transcript assembly and abundance estimation using Cufflinks v2.2.1 [24].

The transcript-level relative transcript abundances were measured in FPKM (Fragments Per Kilobase of exon per Million fragments mapped) using Cufflinks. We performed the statistical analysis to find differentially expressed genes (DEG). Filtered data were log2-transformed and subjected to quantile normalization. For DEG set, Hierarchical clustering analysis was performed using complete linkage and Euclidean distance as a measure of similarity. Gene-enrichment and functional annotation analysis for significant gene list was performed using Gene Ontology (http://geneontology.org/). We used multidimensional scaling (MDS) method to visualize the similarities among samples. We applied to the Euclidean distance as the measure of the dissimilarity.

### Real-time polymerase chain reaction

The cDNA was reverse-transcribed from total RNA using a High-Capacity cDNA Reverse Transcription Kit (Applied Biosystems, Foster City, CA, USA) according to the manufacturer’s instructions. Synthesized cDNA was denatured at 95°C for 10 min and amplified using SYBR Premix Ex Taq II (Takara, Kusatsu, Japan) on an Applied Biosystems 7500 Real-Time PCR System cycler, with 40 cycles of 95°C for 15 s and 60°C for 1 min. All data were acquired as ΔCt values and automatically converted to double delta Ct (ΔΔCt) values by 7500 software (Applied Biosystems). The value of 2−ΔΔCt was calculated to obtain expression fold-change data. Primers specific for CD86, CD40, IFR4, CCR7, FcεR1α, CSF1R, CD163, and CD117 (S1 Table) were used.

### Mixed lymphocyte reaction

For the preparation of allogeneic T cells, splenocytes were isolated and incubated for 2 h to remove attached cells. Floating splenocytes were harvested and labelled with carboxyfluorescein succinimidyl ester (CFSE; Invitrogen). CFSE-labelled allogenic splenocytes were co-cultured with APCs (MHCII^high^ and MHCII^low^ cells) for 5 days. Then, 10^5^ splenocytes were mixed with APCs according to the desired APC:splenocyte ratio (1:2, 1:6, 1:18, 1:54 and 1:162) in 96-well U-bottom plates. For gating proliferating population, CFSE-stained splenocytes was checked their proliferation at 24h and confirmed without proliferation (data not shown). T cell proliferation was examined using flow cytometry and analyzed by FlowJo software.

### Phagocytosis assay

The cells were seeded at 2 × 10^5^ cells and incubated with latex beads coated with FITC-labelled rabbit IgG (Cayman Chemical, Ann Arbor, MI, USA) for 30 min, 1 h, 2 h, 3 h, and 4 h in 96-well U-bottom plates. To distinguish cells that were phagocytosed from those simply binding to the beads at the surface, a short (1–2 min) incubation with Trypan Blue dye quenching solution, followed by a wash with assay buffer, was used to quench the surface FITC fluorescence. Phagocytosed cells were detected using flow cytometry and analyzed by FlowJo software.

## Results

### Heterogeneity of the GM-CSF-derived BMDCs

**Fig 1:**
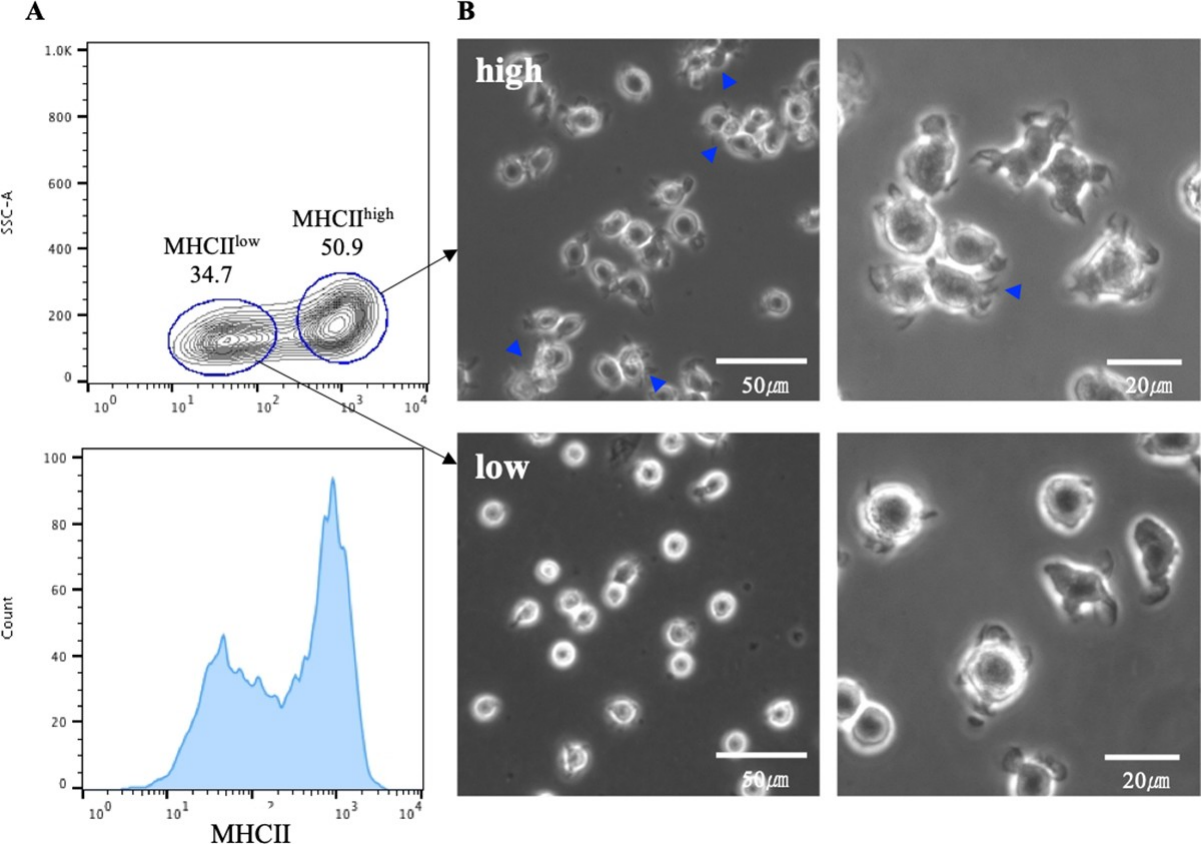
Two distinct populations developing in GM-CSF culture. For *in vitro* generation of dendritic cells, bone marrow cells from gnotobiotic miniature pigs were cultured with GM-CSF for 10 days. (A) After GM-CSF culturing, MHCII expression was confirmed using flow cytometry, and two distinct populations were sorted based on differential expression of MHCII (MHCII^high^ and MHCII^low^). Allophycocyanin (APC)-conjugated goat anti-mouse IgG was used as secondary antibody. (B) The morphology of MHCII^high^ and MHCII^low^ cells was observed using an AxioVert200 inverted microscope at 10×, 20×, and 40× magnification. The blue arrow heads denote cluster formation.

To generate BMDCs *in vitro*, BMCs were obtained from 3-week-old gnotobiotic miniature pigs and cultured with GM-CSF supplementation. To enrich BMDCs, we harvested non-adherent cells from the GM-CSF culture and confirmed the MHCII expression of these cells. The non-adherent cells were comprised of two distinct populations (MHCII^high^ and MHCII^low^) based on the MHCII expression (Fig 1A). Adherent cells were mainly composed of MHCII^low^ population (S1 Fig). We employed FACS sorting to purify MHCII^high^ and MHCII^low^ populations that could not be distinguished by adhesion properties. In the two populations, differences in morphology were observed; the MHCII^high^ cells had a more dendritic morphology, and showed cluster formation, relative to the MHCII^low^ cells (Fig 1B). Thus, MHCII^high^ cells were more representative of DC-like morphology than MHCII^low^ cells. From these results, two populations were isolated from non-adherent cells from porcine BMDC cultures, based on different expression levels of MHCII, in a similar manner to murine cells.

### Surface marker expression levels of MHCII^high^ and MHCII^low^ cells

**Fig 2:**
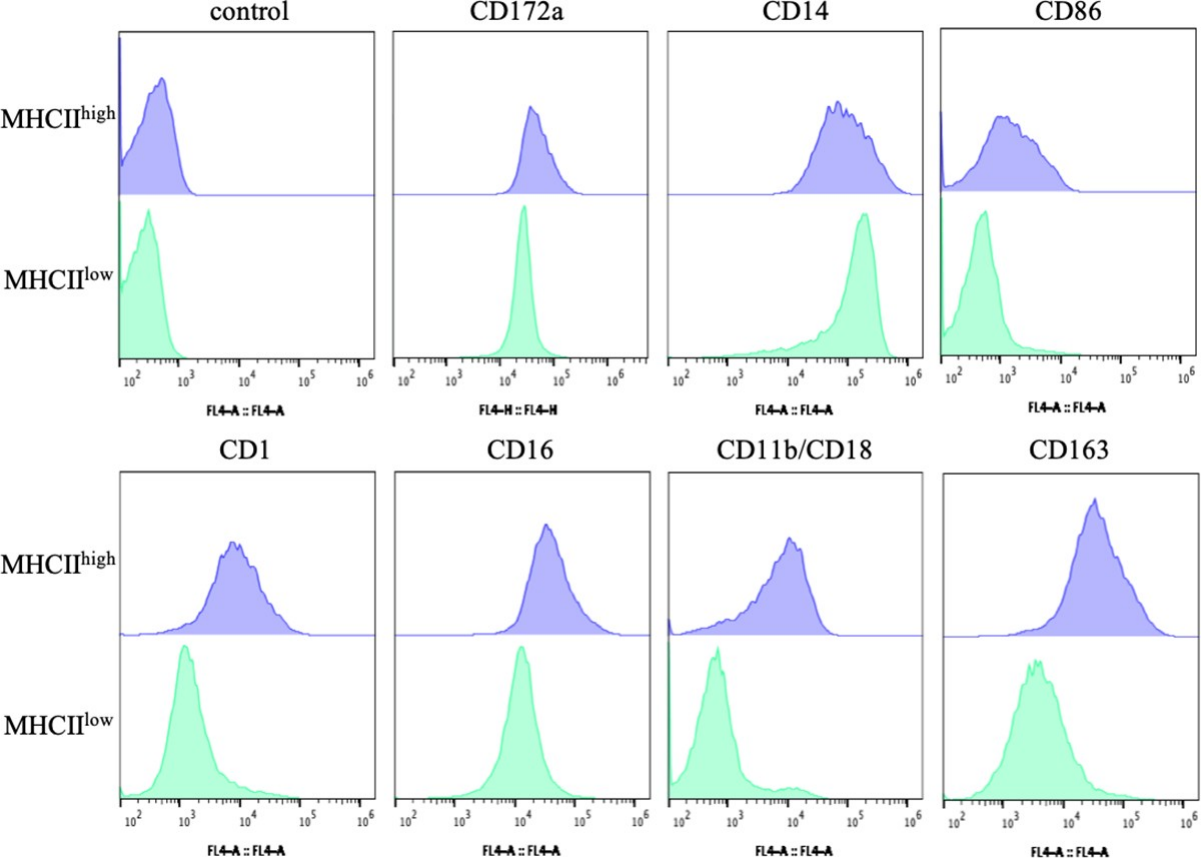
The phenotype of MHCII^high^ and MHCII^low^ cells in GM-CSF culture. The phenotype of cells was analyzed for the expression of the markers of interest by flow cytometry. MHCII^high^ and MHCII^low^ cells were sorted by flow cytometry using fluorescein isothiocyanate (FITC)-conjugated goat anti-mouse IgG as secondary antibodies. The blue-filled histogram shows the MHCII^high^ population and the green-filled histogram shows the MHCII^low^ population. The control represents cells stained only with allophycocyanin (APC)-conjugated goat anti-mouse IgG as secondary antibodies.

Because there were differences in MHCII expression and morphology, MHCII^high^ and MHCII^low^ cells were sorted to confirm their different phenotypes (Fig 2). We examined CD86, CD1, CD16, CD11b/CD18, CD172a, CD14, and CD163 to clearly define distinct populations. The MHCII^high^ and MHCII^low^ cells expressed CD172a and CD14, indicating that they both differentiated into myeloid lineages. We observed high expression of the porcine DC markers, CD86, CD1, and CD16, in MHCII^high^ cells, and low expression in MHCII^low^ cells. Complement receptor CD11b/CD18 and scavenger receptor CD163, which are highly expressed on activated myeloid cells, were found to be highly expressed on MHCII^high^ cells, but they showed very low expression on MHCII^low^ cells. The immature DC phenotype involved intermediate or low expression of MHCII and costimulatory molecules such as CD86, together with high expression of CD14. These results suggested that MHCII^low^ cells resembled immature DCs, and MHCII^high^ cells underwent spontaneous maturation and expressed higher amounts of the same markers.

### Gene expression patterns of MHCII^high^ and MHCII^low^ cells

**Fig 3:**
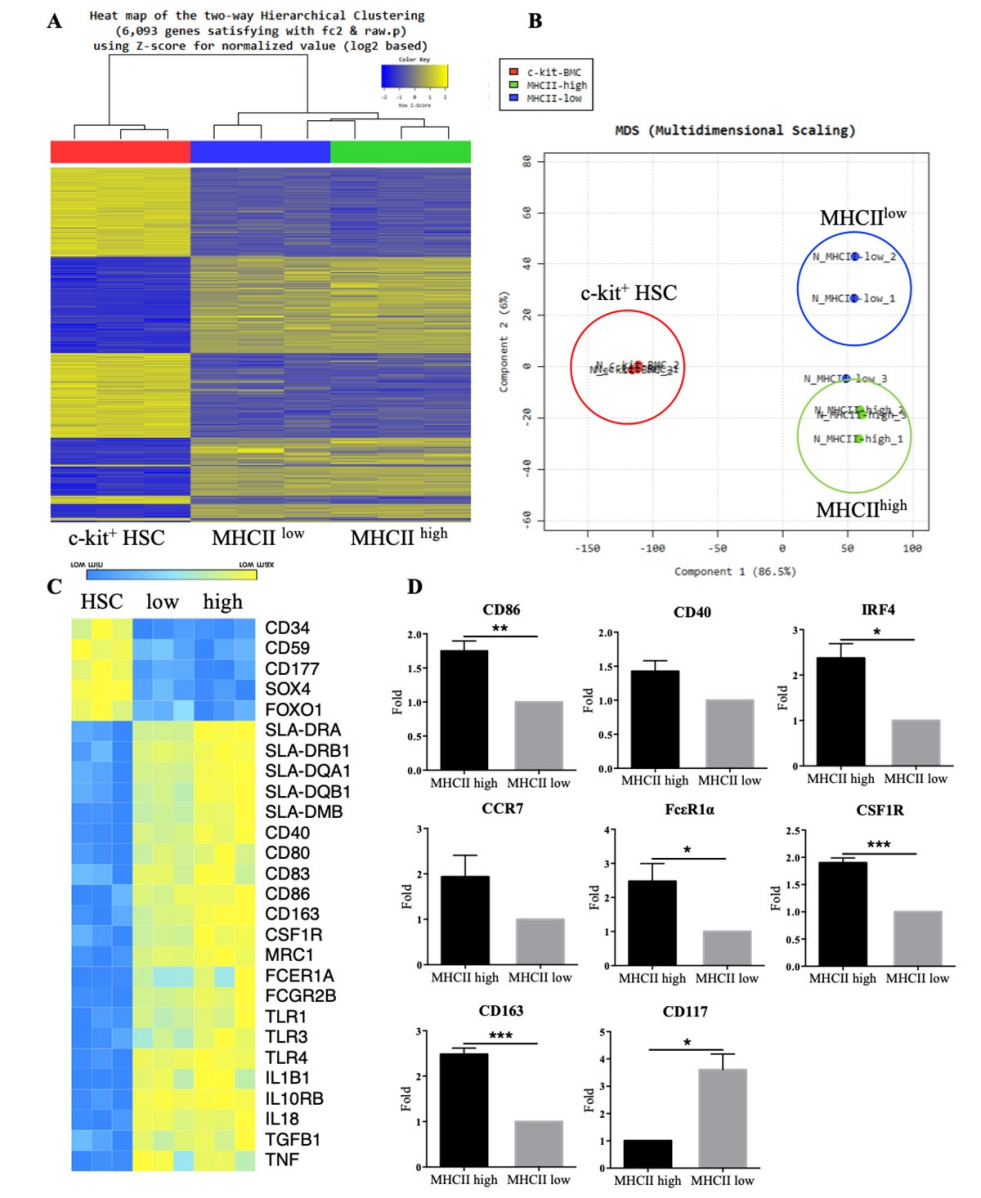
Transcriptional profiling of MHCII^high^ and MHCII^low^ cells in GM-CSF culture. (A) A heat map of hierarchical clustering shows significant transcripts in three independent samples of hematopoietic stem cells (HSCs), MHCII^high^ cells, and MHCII^low^ cells, based on the Euclidian distance with the complete linkage method. (B) A multidimensional scaling plot shows the relationships of HSCs, MHCII^high^, and MHCII^low^ cells in the total gene expression map shown in (A). Each dot represents data from one animal. (C) Heat map of selected transcripts in three independent samples of MHCII^high^ and MHCII^low^ cells. (D) The expression levels of the indicated DC signature genes were analyzed by qPCR. Data are expressed as the mean ± SEM, derived from multiple tests (n = 3). *P < 0.05; **P < 0.005; ***P < 0.0005.

For gene expression analysis, we sorted c-kit^+^ HSCs from BMCs and the two populations (MHCII^high^ and MHCII^low^ cells) described in Fig 1A, followed by mRNA sequencing. Using gene hierarchical cluster mapping and MDS analysis, each cell type from the three different piglets clustered together, confirming that the transcription profiles of each cell type were similar (Fig 3A and 3B). We then confirmed that 368 genes were differentially expressed between MHCII^high^ and MHCII^low^ cells. However, the MHCII^high^ and MHCII^low^ cell populations were close together, suggest that these they are not the completely separated as distinct cell type unlike what has been described for mice and humans [8, 9]. We therefore hypothesized that MHCII^high^ and MHCII^low^ cell populations were comprised of BMDCs in distinct maturation states, as opposed to different cell types being present.

To further explore this possibility, DC-related gene expression patterns were investigated in each cell type. Transcriptome analysis revealed that MHCII (SLA-DR) and costimulatory molecules (CD40, CD80, CD83, and CD86) were highly expressed in MHCII^high^ cells that were induced during BMDC differentiation (Fig 3C). In addition, receptors (CD163, MRC1, FCER1A, FCGR2B, and TLRs) involved in the DC innate immune response were also highly expressed in MHCII^high^ cells. MHCII^low^ cells expressed more CD34, CD59, CD177, Sox4, and Foxo1, which showed the highest expression levels in HSCs. These data showed that MHCII^high^ cells highly expressed genes related to the DC signature and innate immune response, whereas, genes enriched in HSCs were found to be expressed more in MHCII^low^ cells than MHCII^high^ cells.

To determine the difference between MHCII^high^ and MHCII^low^ cells, DC-related gene expression patterns were validated by qPCR. As expected, the CD86 and CD40 costimulatory molecules were expressed at high levels by MHCII^high^ cells, and to a lesser extent by MHCII^low^ cells (Fig 3D). In addition, the IRF4 transcription factor, which controls the development of BMDCs falling within the mature gate, was highly expressed on MHCII^high^ cells compared to MHCII^low^ cells. MHCII^high^ cells also expressed higher amounts of CCR7, FcεR1α, CD163, and CSF1R, whereas MHCII^low^ cells showed higher expression of CD117. Consistent with these results, activated DC-related genes were highly expressed on MHCII^high^ cells compared to MHCII^low^ cells. Therefore, we assumed that the two populations (MHCII^high^ and MHCII^low^ cells) had cell-to-cell variations that were the result of different states of BMDC maturation.

### Functions of MHCII^high^ and MHCII^low^ cells

**Fig 4:**
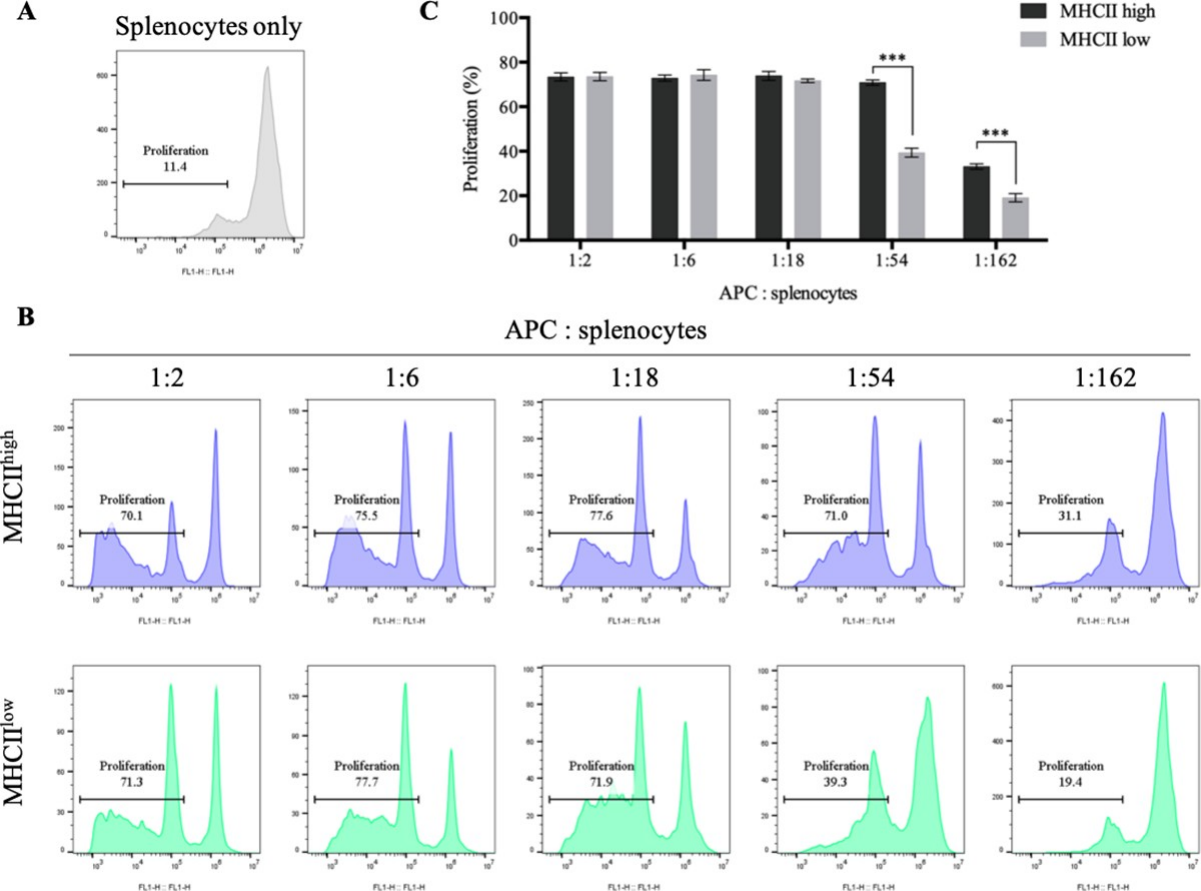
Mixed lymphocytes reaction of MHCII^high^ and MHCII^low^ cells. (A) Splenocytes stained with carboxyfluorescein succinimidyl ester (CFSE) cultured alone for 5 days and analyzed their proliferation. (B, C) CFSE-labelled allogeneic splenocytes were cultured with MHCII^high^ and MHCII^low^ cells at the indicated ratios for 5 days. (B) The blue histogram denotes allogenic splenocytes cultured with MHCII^high^ cells, and the green histogram shows splenocytes cultured with MHCII^low^ cells. The gate indicates the percentage of proliferated T cells.

To examine the ability of DCs that can stimulate T cells as professional APCs, the mixed lymphocyte reaction was conducted using allogeneic splenocytes co-cultured with sorted MHCII^high^ and MHCII^low^ cells (Fig 4). When sufficient APCs were supplied to expand splenocytes (the APC:splenocyte ratio was 1:2 ~ 1:18), the MHCII^high^ and MHCII^low^ cells displayed comparable ability to stimulate T cells. There was no significant difference in the T cell proliferation ability of MHCII^high^ and MHCII^low^ cells. In contrast, when given a lower number of APCs to stimulate T cells (APC:splenocyte ratio, 1:54 ~ 1:162), MHCII^low^ cells were inferior in terms of proliferating T cells compared to MHCII^high^ cells. Accordingly, MHCII^high^ cells showed superior T cell priming capacity compared to MHCII^low^ cells.

**Fig 5:**
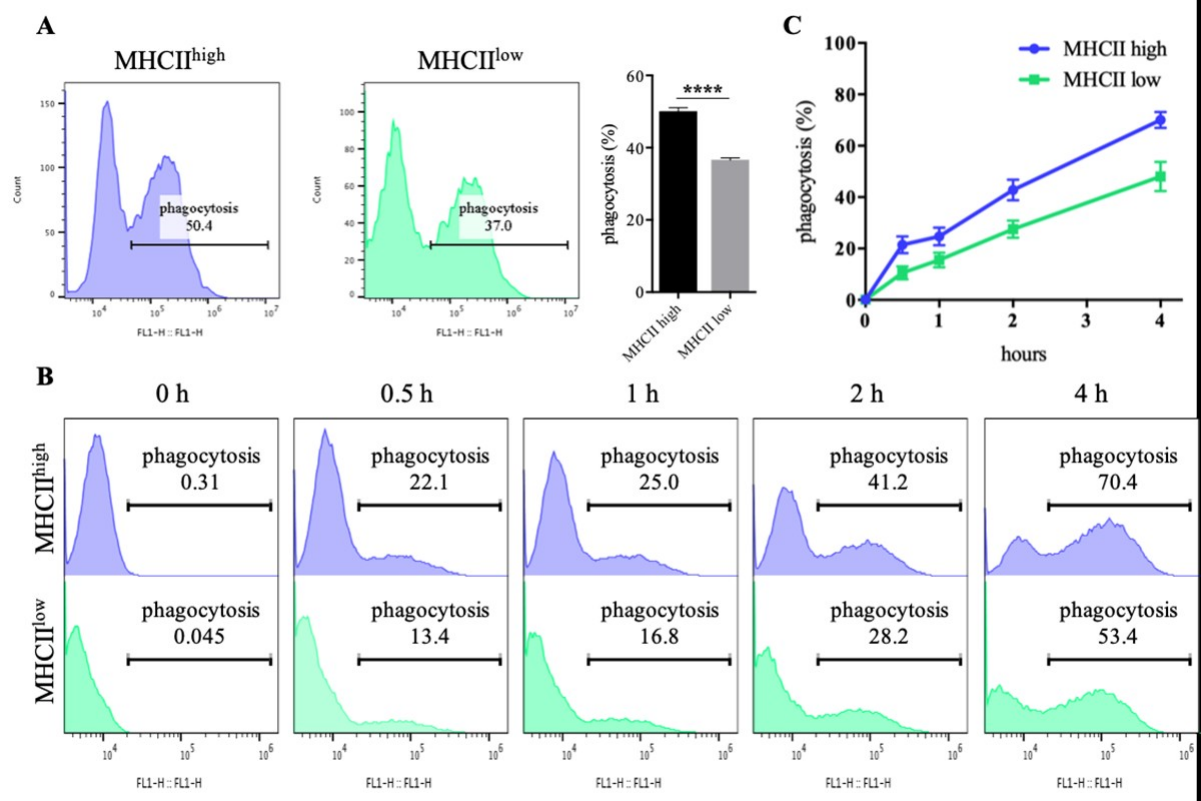
Phagocytosis by MHCII^high^ and MHCII^low^ cells. (A) For analysis of phagocytic bead uptake, MHCII^high^ and MHCII^low^ cells were incubated with fluorescein isothiocyanate (FITC)-tagged latex beads for 3 h. Internalized beads were analyzed by flow cytometry. (B, C) FITC-tagged latex beads were incubated with phagocytes for the indicated times. (B) The blue histogram represents MHCII^high^ cells and the green histogram represents MHCII^low^ cells.

DCs are mononuclear phagocytes; therefore, to analyze phagocytic abilities, MHCII^high^ cells and MHCII^low^ cells were cultured with phagocytic beads (Fig 5). MHCII^high^ cells had more uptake of phagocytic beads during 3 h (Fig 5A; 50% of MHCII^high^ cells and 37% of MHCII^low^ cells). When cells were incubated with phagocytic beads, MHCII^low^ cells were significantly less efficient at phagocytosis, as expected (Fig 5B). Together, these results indicated that MHCII^high^ cells are more functionally activated DCs than MHCII^low^ cells, because they had superior T cell-priming ability and phagocytic ability.

## Discussion

The *in vitro* generation of DCs in culture is advantageous for studying DC biology. In particular, GM-CSF, a hematopoietic growth factor, has been used to supplement BM cultures to generate CD11c^+^ MHCII^+^ cells, which are often termed BMDCs [5]. From the GM-CSF BM culture, DCs have been enriched from non-adherent cells, whereas adherent cells are thought to be macrophages. In addition, the studies of the discrimination of murine BM cultures showed the heterogeneity of GM-CSF-derived non-adherent cells and loosely adherent cells [8, 9]. They suggested that the MHCII^high^ cell population, considered as a DC and MHCII^low^ cell population, actually corresponded to macrophages from murine BM cultures. However, another study showed that GM-CSF culture induced differentiation towards immature and mature cDC2s, which were shown to be efficient at promoting Th17, as well as Th2 immune responses, in a non-adherent population [11].

The pig has been considered an important large animal model, and gnotobiotic miniature pigs are probably the best model for studying immunology [18]. The porcine immune system resembles the human immune system with respect to DC biology, because their gene expression signature for cDC2 is close to the human counterpart [14, 25, 26]. The classical protocols for generating *in vitro* DCs in humans and mice are similar to the porcine method. Although GM-CSF-generated porcine BMDCs have been widely used, the heterogeneity of the cells has not been defined. Considering that murine GM-CSF cultures often provide two populations based on MHCII expression level, we discriminated GM-CSF-grown BMDCs from gnotobiotic miniature pigs based on several criteria.

In this study, we noted heterogeneity in the non-adherent cells from gnotobiotic miniature pigs according to their MHCII expression levels (MHCII^high^ and MHCII^low^ cells). It has been reported that cells developing in porcine GM-CSF culture were also heterogeneous, as in murine cultures. Although both populations showed DC-like morphology, MHCII^high^ cells had a more dendritic morphology, and showed cluster formation, relative to MHCII^low^ cells. The phenotype analysis showed that MHCII^high^ cells displayed a DC-like phenotype that involved CD86^+^, CD1^+^, CD16^+^, CD11b/CD18^+^, CD172a^+^, CD14^low^, and CD163^+^. MHCII^low^ cells also expressed these DC markers; however, they had low expression levels of CD86, CD1, CD16, CD11b/CD18, and CD163, and higher CD14 expression which is downregulated during DC maturation [27]. According to our results, MHCII^low^ cells appear to represent an immature DC phenotype with low expression of MHCII and costimulatory molecules, such as CD86.

In accordance with morphology and phenotype analysis, transcriptome analysis confirmed heterogeneity in BMDC maturation: DC-related genes were highly expressed in MHCII^high^ cells, including costimulatory molecules and innate immune receptors, whereas MHCII^low^ cells showed higher levels of genes mainly expressed on HSCs. The higher expression levels of IRF4 and CCR7 in MHCII^high^ cells supported BMDCs being within the mature gate, as well as the development of subsets into cDC2s [28, 29]. In accordance with, BMDCs under the influence of GM-CSF appeared closer to cDC2s [27]. The MDS analysis revealed the differences between MHCII^high^ and MHCII^low^ cells. It also showed that the few difference between the two cell populations involved the maturation state rather than being due to the presence of distinct cell types. One sample from the MHCII^low^ cell population (MHCII^low^_3) was more close to the MHCII^high^ population in the MDS analysis. It is possible that variations were due to differences between individual samples, or to factors such as variable culture conditions. In further studies, it should be possible to identify their closest relatives *in vivo* by transcriptome analysis of *in vitro*-generated BMDCs from gnotobiotic miniature pigs. Furthermore, our RNA-sequencing data may provide information relevant to the investigation of porcine HSCs and BMDCs.

In addition, MHCII^high^ cells expanded into T cells and phagocytized beads more efficiently than MHCII^low^ cells, with similar gene ontology enrichment of antigen presentation and innate immune receptors. Accordingly, porcine GM-CSF culture preferentially differentiated BMCs into immature (MHCII^low^ cells) and mature (MHCII^high^ cells) DCs.

On the basis of morphological, phenotypical, and gene expression criteria, we classified cell two populations based on MHCII expression; we suggest that the MHCII^high^ and MHCII^low^ populations can be best-classified by their maturation stage. Therefore, this study might lead to a better understanding of the function of DCs and provides useful information for future studies using porcine BMDCs.

## Supporting information

S1 Fig Adherent cells developing in bone marrow GM-CSF culture. In bone marrow culture induced by GM-CSF, adhrent cells were obtained and confirmed MHCII expression by flow cytometry. Allophycocyanin (APC)-conjugated goat anti-mouse IgG was used as secondary antibody.
(DOCX)

S1 Table Primers used in quantitative RT-PCR. (DOCX)
