# Supplementary material for: Heterogeneity of porcine bone marrow-derived dendritic cells induced by GM-CSF
Source: PLoS One. 2019 Nov 5;14(11):e0223590. doi: 10.1371/journal.pone.0223590 (PMC6830806; doi:10.1371/journal.pone.0223590)
Supplement: S1 Fig — In bone marrow culture induced by GM-CSF, adhrent cells were obtained and confirmed MHCII expression by flow cytometry. Allophycocyanin (APC)-conjugated goat anti-mouse IgG was used as secondary antibody. (DOCX) [file pone.0223590.s001.docx]

**Supporting Information**

**
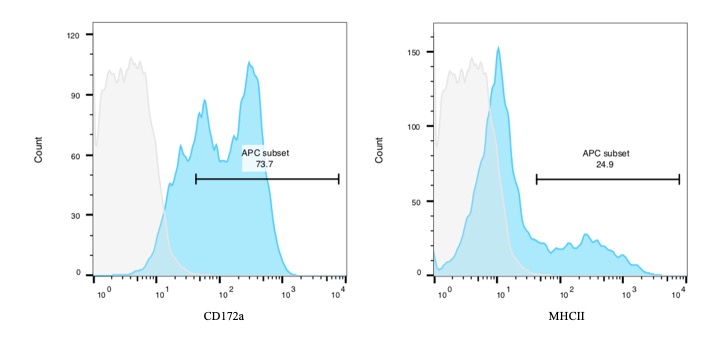
**

**S1 Fig. Adherent cells developing in bone marrow GM-CSF culture.** In bone marrow culture induced by GM-CSF, adhrent cells were obtained and confirmed MHCII expression by flow cytometry. Allophycocyanin (APC)-conjugated goat anti-mouse IgG was used as secondary antibody.
