# Supplementary material for: Heterogeneity of porcine bone marrow-derived dendritic cells induced by GM-CSF
Source: PLoS One. 2019 Nov 5;14(11):e0223590. doi: 10.1371/journal.pone.0223590 (PMC6830806; doi:10.1371/journal.pone.0223590)
Supplement: S1 Table — (DOCX) [file pone.0223590.s002.docx]

**Supporting Information**

**S1 table. Primers used in quantitative RT-PCR.**

| **Gene** | **Species** | **sequence** | |
| --- | --- | --- | --- |
| CD86 | Pig | Forward | CATCGTCTGTGTCCTGCAAC |
|  |  | Reverse | CACAGGTGGCTTTGCATCTA |
| CD40 | Pig | Forward | TGAGAGCCCTGGTGGTTATC |
|  |  | Reverse | GCTCCTTGGTCACCTTTCTG |
| IRF4 | Pig | Forward | CCGGCCTGTGAAAATGGTTG |
|  |  | Reverse | GGACGTGGTCAGCTCTTTCA |
| CCR7 | Pig | Forward | TCCACGTCTGCAAACTCATC |
|  |  | Reverse | GTCGATGCTGATGCAGAGAA |
| FcεR1α | Pig | Forward | AATTTACAGACCCACAGCCTAGCT |
|  |  | Reverse | TGCTATCGCAGATGTTTCTTGAG |
| CSF1R | Pig | Forward | TGAACGACTCCAACTACATTGTCA |
|  |  | Reverse | TGTAGACGCAGTCGAAGATGCT |
| CD163 | Pig | Forward | CACATGTGCCAACAAAATAAGAC |
|  |  | Reverse | CACCACCTGAGCATCTTCAA |
| CD117 | Pig | Forward | TGGGCTCGAGAAGTCAAGTATTT |
|  |  | Reverse | ATGCCCGGAGAGCATTTTT |
